# A nematode-specific ribonucleoprotein complex mediates interactions between the major nematode spliced leader snRNP and its target pre-mRNAs

**DOI:** 10.1093/nar/gkae321

**Published:** 2024-04-27

**Authors:** Peter Eijlers, Mohammed Al-Khafaji, Eva Soto-Martin, Rotimi Fasimoye, David Stead, Marius Wenzel, Berndt Müller, Jonathan Pettitt

**Affiliations:** School of Medicine, Medical Sciences and Nutrition, University of Aberdeen, Institute of Medical Sciences, Foresterhill, Aberdeen AB25 2ZD Scotland, UK; School of Medicine, Medical Sciences and Nutrition, University of Aberdeen, Institute of Medical Sciences, Foresterhill, Aberdeen AB25 2ZD Scotland, UK; School of Medicine, Medical Sciences and Nutrition, University of Aberdeen, Institute of Medical Sciences, Foresterhill, Aberdeen AB25 2ZD Scotland, UK; School of Medicine, Medical Sciences and Nutrition, University of Aberdeen, Institute of Medical Sciences, Foresterhill, Aberdeen AB25 2ZD Scotland, UK; School of Medicine, Medical Sciences and Nutrition, University of Aberdeen, Rowett Institute, Foresterhill, Aberdeen AB25 2ZD Scotland, UK; School of Biological Sciences, University of Aberdeen, Aberdeen AB24 2TZ Scotland, UK; School of Medicine, Medical Sciences and Nutrition, University of Aberdeen, Institute of Medical Sciences, Foresterhill, Aberdeen AB25 2ZD Scotland, UK; School of Medicine, Medical Sciences and Nutrition, University of Aberdeen, Institute of Medical Sciences, Foresterhill, Aberdeen AB25 2ZD Scotland, UK

## Abstract

Spliced leader *trans*-splicing of pre-mRNAs is a critical step in the gene expression of many eukaryotes. How the spliced leader RNA and its target transcripts are brought together to form the *trans*-spliceosome remains an important unanswered question. Using immunoprecipitation followed by protein analysis via mass spectrometry and RIP-Seq, we show that the nematode-specific proteins, SNA-3 and SUT-1, form a complex with a set of enigmatic non-coding RNAs, the SmY RNAs. Our work redefines the SmY snRNP and shows for the first time that it is essential for nematode viability and is involved in spliced leader *trans*-splicing. SNA-3 and SUT-1 are associated with the 5′ ends of most, if not all, nascent capped RNA polymerase II transcripts, and they also interact with components of the major nematode spliced leader (SL1) snRNP. We show that depletion of SNA-3 impairs the co-immunoprecipitation between one of the SL1 snRNP components, SNA-2, and several core spliceosomal proteins. We thus propose that the SmY snRNP recruits the SL1 snRNP to the 5′ ends of nascent pre-mRNAs, an instrumental step in the assembly of the *trans*-spliceosome.

## Graphical Abstract

**Graphical Abstract:**
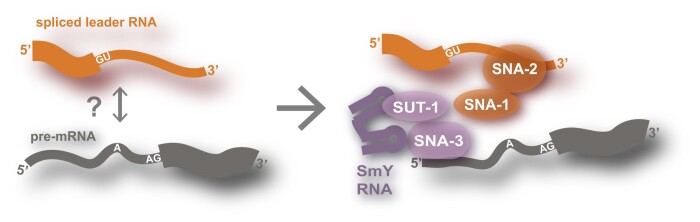

## Introduction

The splicing of pre-mRNAs is a critical step in eukaryotic gene expression. This process involves the coordinated assembly of a complex of up to 200 individual protein and RNA components onto the pre-mRNA, leading to the formation of the ‘spliceosome’, a highly conserved molecular machine required for the removal of introns from pre-mRNAs (1). We have a detailed understanding of the multistep mechanism by which the *cis*-spliceosome recognizes, assembles on, and processes its intron targets (2). These processes require discrete RNA sequence elements—the 5′ splice site, 3′ splice site and the branchpoint sequence—that serve as recruitment points for the assembly of the spliceosome and are instrumental in executing the splicing reaction itself. The 5′ splice site is recognized by the U1 small nuclear ribonucleoprotein particle (snRNP) and the branchpoint sequence and 3′ splice site by SF1 and U2AF protein complexes, respectively. Interactions between these and other spliceosomal components are critical for the subsequent steps in intron splicing (2).

In addition to intron removal, the spliceosome can be used to splice together two distinct RNAs to generate a chimeric mRNA, a process known as *trans*-splicing (3), to distinguish it from *cis*-splicing employed for intron removal. In numerous eukaryotes, spliced leader *trans*-splicing is an important step in pre-mRNA processing (4). It involves the replacement of the 5′ end of a nascent pre-mRNA (termed the ‘outron’) with a ‘spliced leader’ sequence, the splicing reaction taking place between the pre-mRNA and a specialized non-coding RNA termed the spliced leader RNA. Spliced leader *trans*-splicing and *cis*-splicing reactions appear to proceed through essentially the same mechanistic steps (5,6), but assembly of the spliceosome must occur via distinct mechanisms in the two processes. A long-standing but neglected challenge to our understanding of spliced leader *trans*-splicing has been how the spliceosome is assembled onto separate RNA molecules: the 5′ splice site is located on the spliced leader RNA, while the branch point sequence and 3′ splice site are provided by the pre-mRNA. More importantly, the U1 snRNP is not involved in the assembly of the spliceosome in spliced leader *trans*-splicing (5). Thus, an important challenge is characterizing the mechanistic basis of ‘*trans*-spliceosome’ assembly.

We and others have been studying this process in the nematode *C. elegans*, an organism, which, like all characterized nematodes, uses spliced leader *trans*-splicing to sanitize the 5′ untranslated regions (UTRs) of capped pre-mRNAs (7–9). This splicing event is used to process most capped pre-mRNAs in *C. elegans*, and is termed SL1 *trans*-splicing to distinguish it from a second, mechanistically distinct process, termed SL2 *trans*-splicing (10), the latter being used to provide a cap for pre-mRNAs generated from polycistronic RNAs (11,12).

Previous studies have implicated a cohort of nematode-specific proteins in spliced leader *trans*-splicing, these include the two SNA (Small Nuclear RNA Associated) proteins and a paralogue of SNA-1, SUT-1 (13,14). We have shown that loss of these proteins impairs SL1 *trans*-splicing, and defined an additional, previously uncharacterized protein, SNA-3, as an essential component of the SL1 *trans*-splicing machinery (14,15). However, we know little about the molecular functions of these molecules.

SNA-1 and SNA-2 appear to be components of the SL1 snRNP making them well placed for being involved in the assembly of the *trans*-spliceosome. SNA-3 was identified through its interactions with the SL1 snRNP, but it is also associated with the CBC-ARS2 complex. This interaction is striking given the role of this latter complex as a platform for recruiting molecules that determine the fate of RNA polymerase II transcripts (16), and specifically because of its role in promoting splicing (17). SNA-3 also interacts directly with SUT-1 and is essential for the recruitment of SUT-1 to the nucleus. Taken together, our previous work suggested that SNA-3 and SUT-1 form a complex that has dynamics distinct from the SL1 snRNP (15).

A small family of short, non-coding RNAs, termed SmY RNAs (13,18), also appear to be linked to spliced leader *trans*-splicing (13). SmY RNAs are similarly nematode-specific but are detectable in only three of the five nematode clades (Clades III, IV and V), with the eponymous SmY RNA first being identified in Ascaris *in vitro* splicing extracts (6,18). SmY RNAs have a characteristic secondary structure consisting of two stem-loops flanking an Sm binding site. The first stem loop shows extensive sequence conservation suggestive of an important functional motif (18). The functional significance of SmY RNAs is unknown.

In the current study, we report a breakthrough in our understanding of the mechanism of nematode SL1 *trans*-splicing, showing that SNA-3 acts to recruit the SL1 snRNP, facilitating the assembly of the SL1 *trans*-spliceosome. Through its interactions with SUT-1, and the SmY RNAs, we propose that it constitutes the key determinant in the recruitment of SL1 snRNP to pre-mRNAs during the initial stages of spliced leader *trans*-splicing.

## Materials and methods

### Nematode and yeast strains

*C. elegans* strains were grown using standard culture conditions at 20°C, unless otherwise stated. The following previously published strains were used: PE793, *feIs11[P_vit-2_::outron::gfp^M1A^ P_myo-3_::mCherry]* X; PE912, *sut-1 (fe79[sut-1::GFP^3xFLAG])* II; PE918, *snr-2 (fe82[GFP::3xFLAG::snr-2])* I; and PE975, *uocIs1 II; sna-3 (fe92[sna-3::GFP^3xFLAG]) unc-119 (ed3)* III (15). Strains generated in the current study are detailed below.

**Table:** 

Strain	Genotype
PHX4275	*smy-7 (syb4275) feDf7 feDf8 feDf9* IV; *feDf1* V; *feIs11* X
PE853	*feDf7* IV
PE855	*feDf7* IV; *feDf1* V
PE878	*feDf7 feDf8* IV; *feDf1* V
PE881	*feDf7 feDf8 feDf9* IV; *feDf1* V
PE899	*feDf7 feDf8 feDf9* IV; *feDf1* V; *feIs11* X
PE1147	*sut-1(tm3079)* II; *smy-7(syb4275) feDf7 feDf8 smy-9(fe138) feDf9* IV/*nT1[qIs51] (*IV;V); *feDf1/nT1[qIs51]* (IV;V)
PE1146	*smy-7(syb4275) feDf7 feDf8 smy-9(fe138) feDf9* IV/*nT1[qIs51]* (IV;V); *feDf1/nT1[qIs51]* (IV;V)
PE1038	*uocIs1* II*; unc-119(ed3)* III; *sna-2(fe124[sna-2::GFP^3xFLAG])* IV
PE1152	*smy-7(syb4275) feDf7 feDf8 smy-9(fe138) feDf9* IV/*nT1[qIs51]* (IV;V); *feDf1* V/*nT1[qIs51]* (IV;V); *feEx385[smy-8 (+) myo-2p::dTomato]*
PE1154	*smy-7(syb4275) feDf7 feDf8 smy-9(fe138) feDf9* IV/*nT1[qIs51]* (IV;V); *feDf1* V/*nT1[qIs51]* (IV;V); *feEx387[smy-10 (+) myo-2p::dTomato]*
PE1162	*smy-7(syb4275) feDf7 feDf8 smy-9(fe138) feDf9* IV; *feDf1* V; *feEx385[smy-8 (+) myo-2p::dTomato]*
PE1212	*smy-7(syb4275) feDf7 feDf8 feDf9* IV; *feDf1* V; *feIs11* X; *feEX403[smy-8 (+) myo-2p::dTomato]*
PE1217	*smy-7 (syb4275) feDf7 feDf8 feDf9 IV; feDf1* V; *feIs11* X; *feEX407[smy-10 (+) myo-2p::dTomato]*
PE1220	*cshIs140[rps-28p::TIR1 (F79G)::T2A::mCherry::his-11 + Cbr-unc-119 (+)]* II; *sna-3(fe110[sna-3::mNG^AID::3xFLAG]) unc-119 (ed3)* III; *sna-2 (fe124[sna-2::GFP^3xFLAG])* IV

With the exception of *smy-7(syb4275)*, the *smy* gene deletions were generated using the co-conversion approach outlined previously (19,20), deleting individual genes (*smy-7* and *smy-9*), or clusters of genes (*smy-1*, *-2*; *smy-3*, *-4*, *-5*, *-6*; *smy-8*, *-10*; and *smy-11*, *-12*), by using guide RNAs on either side of the targeted locus. PHX4275 was generated by SunyBiotech. Oligonucleotides used to generate guide RNA constructs and to screen for gene deletions by PCR are given in Supplementary Table S1. GFP-tag knock-in strains were generated as described previously, with the details of the plasmids involved described in Supplementary Table S1 (21).

### Transgenic rescuing assays

Wild-type amplicons containing either *smy-8* or *smy-10* were PCR amplified (see Supplementary Table S1), and the resulting amplicons were cloned into pCR4-TOPO (Thermo-Fisher) and verified by Sanger Sequencing (Eurofins). Worms were co-injected with plasmid pMK78 (*smy-8*) or pMK81 (*smy-10*), and p*myo-2p*::dTomato (a generous gift from Rik Korswagen, Hubrecht Institute) and transgenic lines established as previously described (22,23).

To assess rescue of the larval lethality shown by the compound *smy* loss of function mutant, individual transgenic, non-GFP offspring of PE1154 or PE1162 animals were picked as L1 larvae and their ability to reach self-fertile adulthood was scored. Rescue of the SL1 *trans*-splicing defects was assayed using the previously described chromosomally integrated SL1 *trans*-splicing reporter gene (14). We measured the pixel intensity of GFP fluorescence in adult transgenic offspring of PE1212 or PE1217 compared to their non-transgenic siblings using Fiji (24).

### Fluorescent microscopy

Worms were mounted in 5 μl M9 supplemented with 10 mM sodium azide on 5% agar pads. Images were obtained using a Zeiss Axioplan 2, equipped with a Hamamatsu Orca ER camera.

### Preparation of *C. elegans* embryo extracts

*C. elegans* embryo extracts were prepared essentially as described, with some modifications (15,25) (see also Supplementary Methods). RNase treatment of extracts was essentially done as described (15) (see also Supplementary Methods). Where indicated, embryos were treated with 50 μM 5-Ph-IAA-AM dissolved in DMSO or control-treated with DMSO for 1 h at room temperature (26).

### Immunoprecipitation of proteins

Immunoprecipitations of GFP-tagged proteins were performed in triplicates or quadruplicates using anti-GFP nanobody coupled agarose beads and control agarose beads (GFP-Trap and control agarose beads, Chromotek GmbH), essentially as described previously (15) (see also Supplementary Methods).

### Protein analysis by LC–MS/MS and differential protein expression analysis

Proteomic analysis was carried out as previously described (15,27). Raw data files were processed using MaxQuant version 1.6.5.0 (28), as previously described (15), using the *C. elegans* reference proteome UP000001940 downloaded on 24 August 2021 (see also Supplementary Methods). Data analysis was done using Perseus version 1.6.5.0 (29). Relative protein abundance was estimated using the total protein approach, which involves comparing LFQ intensities for individual proteins determined using MaxQuant to the total signal of the sample followed by division by the molecular weight of the identified proteins (30). Relative protein abundance is expressed as a proportion of the GFP-tagged protein ± standard deviation.

### RNA immunoprecipitation sequencing (RIP-Seq)

Immunoprecipitations were performed in quadruplicate using either anti-GFP nanobody coupled agarose beads or control agarose beads as described previously for protein immunoprecipitations (15). Library preparation using the Diagenode D-Plex Small RNA-seq Kit for Illumina. The kit is designed to capture total RNA, including small RNAs. The basic steps include poly(A) tailing and template switching to facilitate library preparation from low RNA inputs. There was no size selection as we wanted to be certain to capture both mRNAs and small nuclear RNAs such as SL RNAs, U snRNAs and SmY RNAs. Library sequencing was performed on an Illumina NextSeq500 high-output v2.5 flow cell, producing 76-bp single end reads, at the Centre for Genome Enabled Biology and Medicine (CGEBM; University of Aberdeen) (see also Supplementary Methods).

### RIP-Seq peak calling

Genome-wide peaks of SNA-3 and SUT-1 RIP-Seq read alignments were identified on each strand independently using MACS2 2.1.2 (31), disabling the ChIP-Seq-specific coverage model (–nomodel –shift 0 –extsize 75), retaining duplicate reads and calling narrow peaks (–min-length 10 –max-gap 10) against local background lambda estimated in 75 and 100 bp windows from matched control libraries prepared using agarose beads only (FDR ≤ 0.05). Each peak was assigned to the nearest downstream or overlapping gene using BEDTOOLS CLOSEST 2.30.0 (32). The peaks were post-processed in R 3.6.1 (https://www.R-project.org/), trimming peak shoulders either side of the peak summit where read coverage dropped below 50% of the summit coverage. To identify possible common sequence motifs present in the SNA-3 and SUT-1 read peaks, the nucleotide sequences enclosed by the shoulders of each peak were extracted from the genome using BEDTOOLS GETFASTA and clustered at 50–90% sequence similarity using VSEARCH 2.15.1 (33).

### Transcription start site identification and quantification

Coordinates of curated transcription start sites (TSSs) were obtained from four independent studies (34–37). Where a study reported multiple TSSs at the same transcript, the TSS with the highest read depth was selected (when necessary, ties were broken by selecting the TSS with shorter distance to the 5′ end of the transcript). The TSS coordinates were lifted to the WBCel235 genome assembly using the UCSC liftOver utility (38). The WBGene ID or transcript codes assigned to the TSSs by the original studies were converted to WBCel235 WBGene IDs using the WormBase Gene Name Sanitizer. For each TSS, the nearest downstream or overlapping gene was obtained using BEDTOOLS CLOSEST 2.30.0 (32) and the TSS was retained if the WBGene IDs matched. Where the gene annotation did not already overlap the TSS, the annotation was extended upstream to ensure that any RIP-Seq reads originating from the TSS would overlap the gene annotation. This did not introduce ambiguity from unwanted overlap between neighbouring gene annotations.

Genome-wide RIP-Seq read alignments were then quantified against all overlapping gene annotations with FEATURECOUNTS 2.0.2 (39), assigning fractional counts to all locations for multimapping reads. Genes that were significantly enriched (log_2_ fold change > 0; FDR ≤ 0.05) in anti-GFP libraries were identified using DESeq2 1.42.0 (40), shrinking fold changes with the *apeglm* method (41). Overrepresentation of GeneOntology (*biological process* ontology) terms and KEGG pathways (*q*-value ⇐ 0.05) among significantly enriched genes was tested using CLUSTERPROFILER 4.10 (42), ignoring GO terms at levels 1–3 and removing redundancy among GO terms via semantic similarity clustering at 70% (43) and retaining the most significant term for each cluster.

### Identification of associations between RIP-Seq peaks, TSSs and transcripts

The nature of the TSS and RIP-Seq peak data meant that we could not determine whether a TSS and a RIP-Seq peak originated from the same transcript. This is further complicated when a gene contains multiple TSSs and/or RIP-Seq peaks. We implemented the following heuristics to identify the most likely associations between RIP-Seq peaks, TSSs and transcripts: (i) for each TSS, we retained the nearest downstream transcripts (measured to the 5′ end of the first CDS annotation for coding transcripts). The TSS was permitted to be at most 50 bp downstream of the 5′ end if the TSS was located within the first CDS; this allowed for rescuing edge cases with potentially incorrect designation of start codons. (ii) To each TSS we assigned the nearest high-quality RIP-Seq peak (read depth ≥ 20 reads, enrichment score ≥ 5, peak width 30–90 bp). (iii) Where this caused a RIP-Seq peak to be assigned to multiple TSSs, we retained the TSS with the smallest distance to the peak. (iv) For each transcript we retained the TSS/peak pair with the smallest distances between the TSS and the peak. Distributions of distances of RIP-Seq peaks to TSSs or 5′ ends of transcripts were inspected and compared against a null distribution derived from randomly generated peaks across the genome using Kolmogorov-Smirnov tests. The randomly generated peaks underwent the same assignment heuristics to TSSs and transcripts as described above for the RIP-Seq peaks. We verified our assignment heuristics by visually inspecting the locations of TSSs, RIP-Seq peaks and gene models in IGV (44) focussing particularly on 98 genes that are expected to be expressed in embryos (Supplementary Table S4).

### Annotation of spliced leader *trans*-splice acceptor sites

Genome-wide information on spliced leader *trans*-splice acceptor sites and exon-exon junctions (location and read depth) was obtained for 17531 genes from (8). Background gene expression was estimated from the median exon-exon junction read depth of the most strongly expressed junction class (constitutive or major, as previously defined (8)). For each focal transcript, we retained the single spliced leader acceptor site with the highest read depth (≥100 reads) that was located any distance upstream or at most 50 bp downstream of the 5′ end of the CDS. We considered the 5′ end of the gene spliced leader *trans*-spliced if the read depth at the acceptor site was similar to the background read depth but not excessively lower. We identified an empirical cut-off by examining the distribution of acceptor:background coverage ratios genome-wide, which indicated a median ratio of 11.3% and a lower quartile ratio of 2.9%. We thus enforced a minimum acceptor:background ratio of 2.9% to classify a gene as *trans*-spliced, and then compared the proportions of *trans*-spliced genes with our predicted proportions based on the location of the RIP-Seq peak relative to the 5′ CDS end, using chi-square tests.

### Monitoring SL1 *trans*-splicing using quantitative PCR

N2, PE853, PE855, PE899 and PHX4275 animals were synchronized by bleaching and then grown to gravid adulthood on NGM plates seeded with OP50. Animals were harvested by washing with M9 buffer, pelleted, and resuspended in 1 ml TriZol, flash frozen in liquid N2, and stored at −80°C. Total RNA was isolated using the PureLink RNA Mini kit (Life Technologies) with modifications for TRIzol treated samples and DNAse treatment as described by the manufacturer. 1 μg total RNA was reverse transcribed to cDNA using oligo (dT) Primers (Promega) and Superscript III Reverse Transcriptase (Invitrogen) in a volume of 20 μl, according to the manufacturer's instructions. The qPCR assays, including primer sequences and Universal ProbeLibrary probes (Roche) used to monitor SL1 *trans*-splicing of *rps-3* transcripts, were performed as described previously (14), and analysed using a LightCycler 480 (Roche) with proprietary software (LightCycler® 480 Software release 1.5.1.62 SP3).

Animals were grown as biological replicates in three independent cultures, and CT values for each biological replicate were calculated as the average of three technical replicates. Inhibition of SL1 *trans*-splicing is shown as ΔCT values derived for each biological replicate standardized by comparison to the wild type (N2) (ΔΔCT) (45). Note, additional technical information is provided in Supplementary Methods.

## Results

### SNA-3 and SUT-1 proteins are part of an snRNP distinct from the SL1 snRNP.

We have previously shown that SUT-1 protein interacts with SNA-3 protein, a novel *trans*-splicing factor that interacts with the CBC-ARS2 complex and other proteins implicated in RNA metabolism (15). To validate this interaction, we performed immunoprecipitations using a strain that expresses SUT-1 C-terminally fused to GFP, with the expectation that SUT-1::GFP would associate with the same set of proteins as SNA-3. Our previous work indicated that SUT-1 and SNA-3 form a complex that is distinct from, but interacting with the SL1 snRNP, so to confirm the specificity of the SUT-1 interactions we carried out similar immunoprecipitations with a strain expressing endogenous SNA-2 tagged at the C-terminus with GFP.

**Figure 1.**
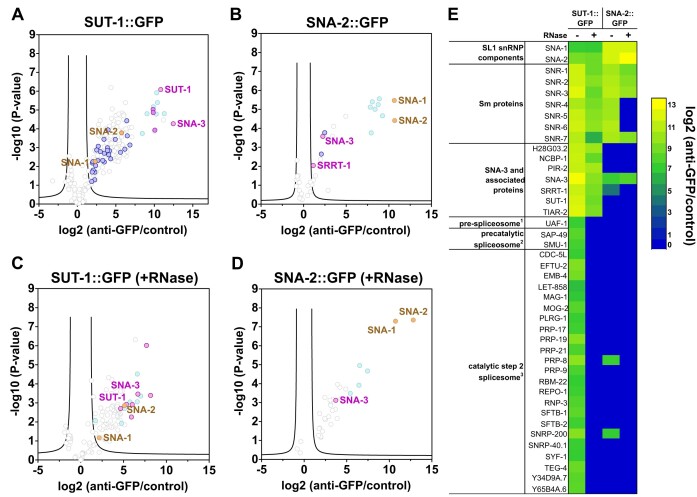
SUT-1 and SNA-3 are components of a complex that interacts with but is distinct from the SL1 snRNP. Proteins enriched by co-immunoprecipitation with SUT-1::GFP (**A, C**) and SNA-2::GFP (**B, D**) from extracts treated without (A, B) and with RNase (C, D). Immunoprecipitations were performed on embryonic extracts using anti-GFP nanobodies coupled to agarose beads, or control agarose beads. Immunoprecipitations were performed in triplicate, and the immunoprecipitated proteins analysed by label-free quantification, using MaxQuant and Perseus software. Graphs show enrichment in anti-GFP nanobody immunoprecipitations compared to bead only controls (x-axis) and the false discovery rate (y-axis). Proteins that were significantly enriched in previously performed SNA-3 immunoprecipitations are indicated in magenta (15). SNA-1 and SNA-2, specific components of the SL1 snRNP, are in gold. The Sm proteins associated with snRNA are indicated in light blue; splicing proteins, dark blue. (**E**) Heatmap summarising the enrichment of SL1 snRNP components SNA-1 and SNA-2, SNA-3 and associated proteins and Sm proteins. Spliceosome components were identified using the gene ontology annotation on UniprotKB (49) and then refined through the identification of human orthologues. Grouping is based on gene ontology (cellular component) terms of human proteins, and was simplified using terms that reflect the various spliceosomal complexes. GO terms pre-spliceosome (1) (GO:0071004), precatalytic spliceosome (2) (GO:00710050) and catalytic step 2 spliceosome (3) (GO:0071013) correspond to spliceosomal complexes A, B and C, respectively (2,15,49,50). For complete lists of identified proteins see Supplementary Table S2.

SUT-1::GFP immunoprecipitations from extracts treated without and with RNase showed robust associations with SNA-3, and the CBC-ARS2 components NCBP-1, NCBP-2 and SRRT-1. Other highly enriched proteins included TIAR-2, PIR-2, and H28G03.2, and Sm proteins (Figure 1A, C, E, Supplemental Table 2). Consistent with a direct interaction between SUT-1 and SNA-3, these proteins are similarly enriched in immunoprecipitations of SNA-3::GFP (15). We also detected enrichment of the SL1 snRNP components, SNA-1 and SNA-2 in SUT-1::GFP immunoprecipitations (Figure 1A, C, E). In addition, we observed enrichment of splicing factors. Most of these are linked to the catalytically active spliceosome, but a few of them are involved at earlier stages of the reaction (Figure 1A, E). Treatment of the extract with RNase before immunoprecipitation prevents the enrichment of the splicing factors, compatible with an indirect interaction mediated by RNA (Figure 1C, E). In SNA-2::GFP immunoprecipitations, we detected enrichment of the SL1 snRNP components SNA-1 and Sm proteins, and of the splicing factors PRP-8 and SNRNP-200, which are also found in SUT-1::GFP immunoprecipitations (Figure 1B, E). We clearly see that the enrichment of SNA-3 (and SRRT-1) is much less than enrichment of the SL1 snRNP components. The co-precipitation of SNA-3 with SNA-2 is resistant to pretreatment of the extract with RNase (Figure 1D, E). This is in contrast to the interactions of SNA-2 with the two splicing factors PRP-8 and snRNP-200 and with SRRT-1, which were sensitive to RNAse treatment.

The generally low enrichment of SNA-1 and SNA-2 in SUT-1::GFP immunoprecipitations fits with the view that SNA-3/SUT-1 form a complex that displays transitory interactions with the SL1 snRNP (Figure 1A, E). This is further underlined by considering the SNA-2::GFP immunoprecipitations (Figure 1B, E), where we clearly see that the enrichment of SNA-3 (and SRRT-1) is much less than the components of the SL1 snRNP - SNA-1 and the Sm proteins. And indeed SUT-1 is not enriched at all.

If there are two distinct complexes involved in SL1 *trans*-splicing, then we would expect this to be reflected in the stoichiometry of the immunoprecipitation enrichments. To crudely compare the stoichiometries of the two complexes, we estimated the relative abundance of each protein in the SUT-1::GFP and SNA-2::GFP immunoprecipitations using the total protein approach for quantitation of proteomic data (Figure 1A, B) (30). In SNA-2 immunoprecipitations, the average abundance of SNA-1 is 107.5% (±12.6%) of SNA-2 abundance, while the abundance of SNA-3 is 0.06% (±0.01%) of SNA-2, and SUT-1 was not detected. In contrast, in SUT-1 immunoprecipitations, SNA-3 abundance is 94.7% (±7.4%) of SUT-1 while the abundance of SNA-2 and SNA-1 are 1.6% (±0.1%) and 0.5% (±0.1%) of SUT-1, respectively. These estimates are thus compatible with two distinct complexes, one containing SNA-1 and SNA-2, and the other SUT-1 and SNA-3, though as we noted previously (15), this may simply reflect the steady state levels of the complexes and does not rule out dynamic interactions not captured by this technique.

### SNA-3 and SUT-1 interact with a nematode-specific family of non-coding RNAs

**Figure 2.**
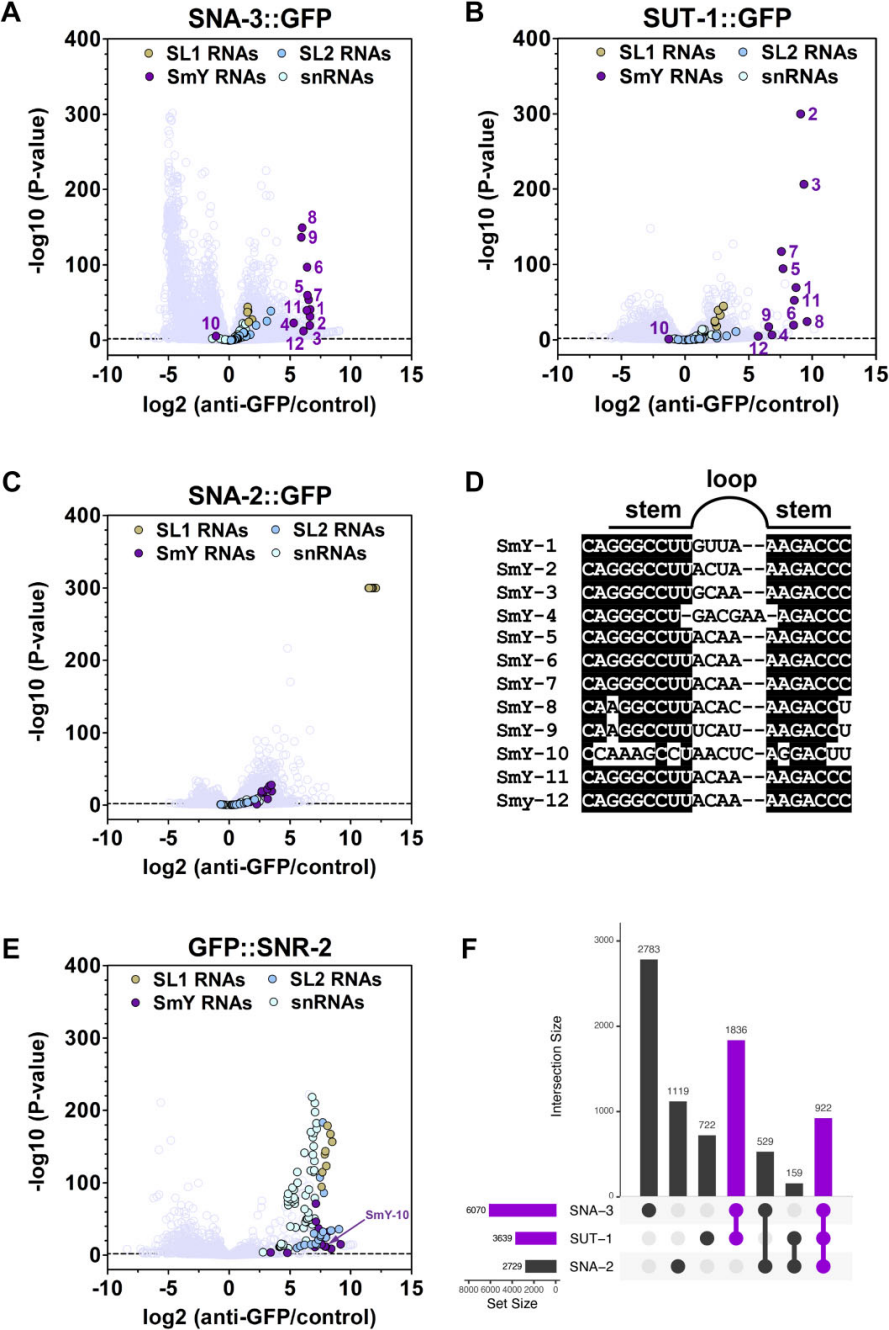
SUT-1 and SNA-3 preferentially associate with SmY RNAs. (A–C) Enrichment of RNA in immunoprecipitations from embryonic extracts expressing GFP-tagged SNA-3 (**A**), SUT-1 (**B**) or SNA-2 (**C**), as assayed by RIP-Seq. Graphs show RNA enrichment in anti-GFP nanobody immunoprecipitations compared to bead only controls (x-axis) and the P-values for these enrichment signals (y-axis). Values for specific non-coding RNAs are colour coded as indicated. All other RNAs are unshaded. Numbers in A and B indicate each of the twelve distinct SmY RNAs. In (B), the *P*-value for SmY-2 RNA was 0 and in (C), the *P*-value for all SL1 RNAs was 0, and we arbitrarily assigned these RNAs a −log_10_(*P*-value) of 300. Dotted line indicates Benjamini–Hochberg false discovery rate threshold. (**D**) Alignment of the first stem loops for each *C. elegans* SmY RNA. Countershading indicates residues that are conserved in at least 75% of SmY RNAs. Gaps are introduced arbitrarily to align secondary structures. The cartoon above indicates the location of residues with respect to the stem–loop. (**E**) Enrichment of RNA in immunoprecipitations from embryonic extracts expressing GFP::SNR-2 as assayed by RIP-Seq (graph details as for A–C). The signal corresponding to SmY-10 is indicated. (**F**) Intersection plot showing the overlaps between genes whose transcripts are significantly enriched (log_2_ fold change > 0; FDR ≤ 0.05) in immunoprecipitations from embryonic extracts expressing GFP-tagged SNA-3, SUT-1 and SNA-2. See Supplementary Table S3 for detailed RIP-Seq data.

Since it appeared that SNA-3 and SUT-1 were not tightly associated with the SL1 snRNP but are nevertheless involved in spliced leader *trans*-splicing, we sought to better understand their function by investigating the RNAs with which they associate. To identify such RNAs we performed RNA Immunoprecipitation Sequencing (RIP-Seq) using strains expressing GFP-tagged endogenous SUT-1 and SNA-3 proteins (Figure 2A, B). As comparators, we performed RIP-Seq using a strain that expresses SNA-2::GFP (Figure 2C) and one that expresses a GFP-tagged version of the *C. elegans* SmB homologue, SNR-2 (Figure 2E). These analyses revealed striking similarities in the RIP-Seq profiles of SUT-1::GFP and SNA-3::GFP, and showed these to be distinct to that of SNA-2::GFP.

We expected strong enrichment of SL1 RNAs in the SNA-2::GFP immunoprecipitations, and indeed the SL1 RNAs were >2000-fold enriched, while the SmY and snRNAs were at best 3.5-fold enriched (Figure 2C). This is compatible with SNA-2 being a component of the SL1 snRNP, and is in agreement with our earlier findings demonstrating that SNA-1 associates preferentially with SL1 RNAs (15). Similarly, in the GFP::SNR-2 immunoprecipitations we expected to see enrichment primarily of SL1 RNA, SL2 RNA, SmY RNA and the snRNAs, since all of these RNAs have known Sm protein binding activity, and this is what we observed in the RIP-Seq data (Figure 2E).

In contrast, the most significantly enriched RNAs in the SNA-3/SUT-1::GFP immunoprecipitations belonged to the SmY RNA family (Figure 2A, B). There are twelve distinct *C. elegans* SmY RNAs, each encoded by a different gene. In SNA-3::GFP/SUT-1::GFP immunoprecipitations, eleven of the SmY RNAs were strongly enriched (Figure 2A, B). In contrast, SL1 RNAs (and SL2 RNAs) and snRNAs showed much lower levels of enrichment, again supporting the hypothesis that SNA-3/SUT-1 form a *trans*-splicing complex distinct from the SL1 snRNP.

The one SmY RNA, SmY-10, that is not enriched in SNA-3/SUT-1::GFP immunoprecipitations (Figure 2A, B) is missing the conserved residues that form the first stem–loop motif (Figure 2D). This could indicate that this motif is required for SmY-10 to interact with these proteins, but the loss of SNA-3/SUT-1 association could also be explained if this motif is required for SmY RNP assembly or stability. However, consideration of the GFP::SNR-2 RIP-Seq data shows that the absence of SmY-10 interaction is specific for SNA-3/SUT-1 (Figure 2E; see Supplementary Figure S1 for protein interaction data for GFP::SNR-2). SmY RNAs were identified based on their interaction with Sm proteins (6), and the twelve SmY RNAs possess a canonical Sm-binding site. As predicted, all SmY RNAs, including SmY-10, associated with GFP::SNR-2 (Figure 2E), and SmY-10 is the second most enriched SmY RNA in this analysis. This indicates that SmY-10 RNA is transcribed and assembles into an Sm bound snRNP but is likely non-functional in terms of SL1 *trans*-splicing due to its failure to interact with SNA-3/SUT-1.

Beyond the enrichment of SmY RNAs, we observed transcripts derived from between 2729 and 6070 genes in total were significantly enriched (log_2_ fold change > 0; FDR ≤ 0.05) in each experiment (Figure 2A–C). As expected, these gene sets represent a broad range of physiological processes, and were enriched for only a small number of pathways associated with signal transduction, energy metabolism and nucleic acid processing (Supplementary Table S3). Of the total enriched genes, 19.8–45.8% were unique to each experiment, whereas most genes were shared between at least two experiments (Figure 2F). Importantly, 75.8% of genes associated with a SUT-1 were also associated with SNA-3 (Figure 2F), supporting the hypothesis that SNA-3 and SUT-1 occupy the same complex.

Taken together, our findings indicate that SUT-1 and SNA-3 interact specifically with Sm protein bound SmY RNAs, presumably as components of the SmY snRNP and that the conserved first stem-loop motif is required for this interaction. This further supports the idea that SUT-1 and SNA-3, while they may form transient interactions with the SL1 snRNP, are part of a mechanistically distinct ribonucleoprotein complex.

### SmY RNAs perform an essential function and their loss causes defects in SL1 *trans*-splicing

**Figure 3.**
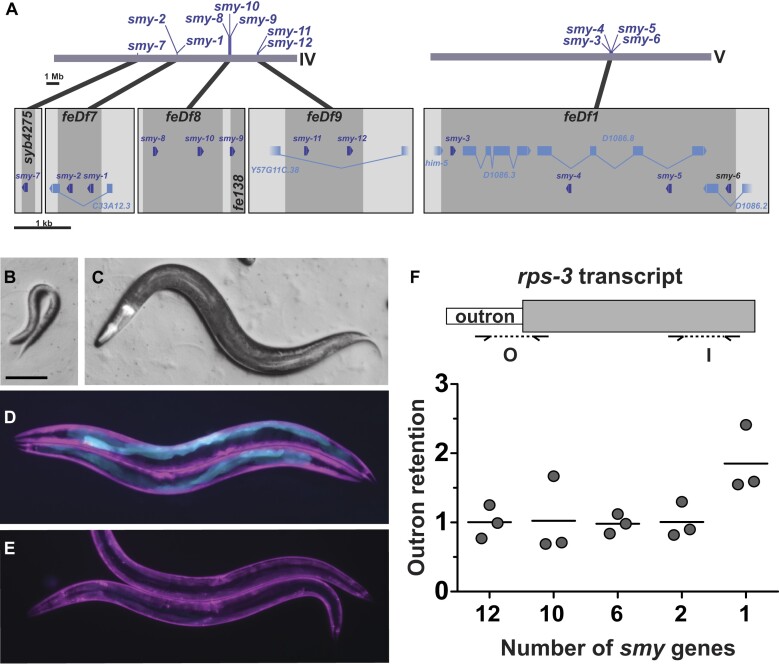
SmY RNAs are essential for postembryonic development, and their loss impairs SL1 *trans*-splicing. (**A**) Schematic of *smy* gene locations on Chromosomes IV and V. Higher magnified views of each locus are shown below. The extent of each deletion mutation is indicated by dark grey shading. (**B**) A homozygous *smy* compound mutant, lacking all twelve *smy* genes. (**C**) Sibling of the animal in panel B - pharyngeal GFP fluorescence indicates that it is heterozygous for the twelve *smy* deletion mutations. Scale bar is 100 μm. (D, E) We previously developed a *C. elegans* strain carrying a *gfp* reporter gene that is expressed in the intestine when SL1 *trans*-splicing is inhibited (14). The *gfp* reporter is strongly activated (D, cyan) in animals lacking eleven of the twelve smy genes, indicating impaired SL1 *trans*-splicing (**D**). Animals with a wild-type complement of *smy* genes show no intestinal GFP (**E**). All worms express mCherry in their body wall muscles (magenta). (**F**) Quantitative RT-PCR measurement of outron retention in *rps-3* transcripts. Schematic shows the location of the primers used to detect outron (O) versus internal (I) regions of the transcripts. Plot shows the ratio of outron to internal amplicon levels for three biological replicates for each genotype—wild type (12 *smy* genes) and mutant strains that retain progressively smaller complements of *smy* genes. Ratios are normalised to wild type using the comparative CT method (45). Horizonal lines indicate mean values.

To determine the functional significance of the SmY snRNAs, we systematically deleted all twelve *C. elegans smy* genes, generating intermediate strains lacking 2 (*smy-1* and *-2*), 6 (*smy-1* to *-6*), 8 (*smy-1* to *-6*, and *smy-8* and *-10*), 10 (all but *smy-7* and *smy-9*) and 11 *smy* genes (all but *smy-9*) *smy* genes (Figure 3A). Animals homozygous for deletion of 10 of the 12 *smy* genes were viable and fertile. However, a strain homozygous for deletion mutations in all but *smy-9* showed significantly reduced viability (Table 1), while deletion of all twelve *smy* genes resulted in fully penetrant larval lethality (Figure 3B, C).

**Table 1.** Loss of *smy* genes compromises viability

Genotype	*smy* genes retained	Viability (%)^a^	*n*
wild type	All twelve	98.2	1073
*feDf7 feDf8 feDf9* IV; *feDf1* V	*smy-7*; *smy-9*	96.9	865
*smy-7(syb4275) feDf7 feDf8 feDf9* IV; *feDf1* V	*smy-9*	18.6	644
*smy-7(syb4275) feDf7 feDf8 smy-9(fe138) feDf9* IV; *feDf1* V	None	0.0	46^b^
*smy-7(syb4275) feDf7 feDf8 smy-9(fe138) feDf9* IV; *feDf1* V; *feEx385[smy-8(+) myo-2p::dTomato]*	*smy-8* ^c^	52.1	535
*smy-7(syb4275) feDf7 feDf8 smy-9(fe138) feDf9* IV; *feDf1* V; *feEx387[smy-10(+) myo-2p::dTomato]*	*smy-10* ^c^	0.0	37

^a^Percentage of animals reaching fertile adulthood.

^b^Offspring of balancer strain: *smy-7(syb4275) feDf7 feDf8 smy-9(fe138) feDf9* IV*/*nT1 [qIs51](IV;V); *feDf1* V/nT1[qIs51](IV;V).

^c^Wild-type copy on extrachromosomal array.

Since many of the *smy* genes lie in introns of, or close to other genes, it was important to confirm that the larval lethal phenotype is due to loss of the *smy* genes and not due to collateral damage of one or more neighbouring genes. We thus performed transgene complementation tests with a wild-type copy of the *smy-8* gene (Table 1). Transgenic *smy-8* was able to rescue the larval lethal phenotype, confirming that the phenotype is caused by loss of SmY RNA function.

Since loss of all 12 *smy* genes can be compensated by expression of an individual *smy* gene, this suggested that most of the *smy* genes may be functionally interchangeable. The one exception would be expected to be SmY-10, which, since it does not interact with SNA-3/SUT-1, should not be able to compensate for compound loss of *smy* gene function. This is indeed the case; our transgenic rescue assay shows that the wild-type *smy-10* transgene fails to rescue the larval lethal phenotype (Table 1). Thus, *smy-10* may be a pseudogene, or it may have acquired a distinct novel function and lost its ancestral activity.

To determine whether SmY RNA function impacted SL1 *trans*-splicing, we examined the effect of loss of *smy* gene function on the activity of our previously devised GFP-based assay for SL1 *trans*-splicing (14). The assay relies on the expression in the adult intestine of an SL1 *trans*-spliced GFP transcript that lacks the normal AUG codon. Instead, an in-frame AUG was placed in the outron, such that impaired SL1 *trans*-splicing leads to accumulation of outron-retaining GFP transcript and hence intestinal GFP fluorescence. We were not able to assay animals lacking all twelve *smy* genes, since complete lack of *smy* genes is larval lethal, and our assay is only active in the intestines of adult worms. However, a strain lacking eleven *smy* genes showed intestinal GFP expression in all animals (*n* ≥ 500; Figure 3D), indicating accumulation of outron retaining *gfp* transcripts consistent with impaired SL1 *trans*-splicing.

As an independent confirmation of the effect of loss of *smy* genes on SL1 *trans*-splicing, we applied our previously described quantitative reverse-transcriptase PCR assay, which examines outron retention in the transcripts of the endogenous *rps-3* gene (14,15). We assayed animals lacking two (*smy-1* and *-2*), six (*smy-1* to *-6*), ten (all but *smy-7* and *smy-9*) and eleven smy genes (all but *smy-9*) (Figure 3F). All genotypes assayed were viable and fertile and loss of up to ten *smy* genes had no detectable effect on *rps-3* outron levels. However, animals lacking eleven *smy* genes displayed a detectable increase in the levels of outron-containing *rps-3* mRNAs, indicating an inhibition of *rps*-3 RNA SL1 *trans*-splicing. It is noteworthy that the eleven *smy* mutant strain, which retains only wild-type *smy-9*, displays partially penetrant larval lethality, showing that the qPCR data correlates with severity of phenotype. Thus, SmY-7 and SmY-9 RNAs together are sufficient to maintain wild-type viability and levels of *rps-3* SL1 *trans*-splicing.

**Figure 4.**
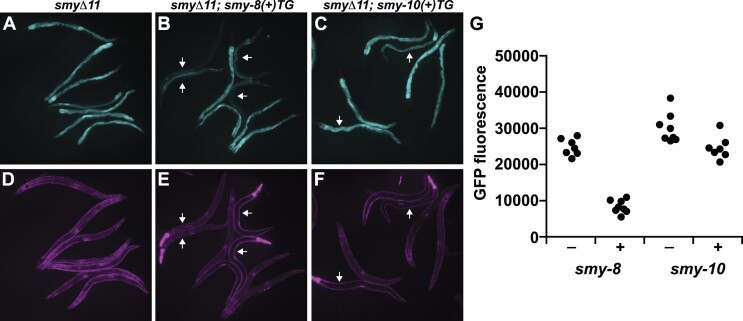
The SL1 *trans*-splicing defect in animals lacking eleven *smy* genes (*smyΔ11*) is rescued by *smy-8* but not *smy-10*. Adult intestinal GFP expression (**A–C**) in animals carrying the SL1 *trans*-splicing reporter (14) indicates reduced SL1 *trans*-splicing. (**D–F**) Constitutive expression of mCherry in the body wall muscle in all worms. The dTomato fluorescence in the pharynx indicates presence of the wild-type *smy-8* or *smy-10* transgenes. Arrows indicate transgenic worms. (**G**) Quantitation of intestinal GFP fluorescence (16-bit pixel scale) of *smyΔ11* compound mutants in the presence (+) or absence (−) of wild-type *smy-8* or *smy-10* transgenes. Each data point represents the average intestinal fluorescence of a single worm.

To further investigate individual *smy* gene function, we assayed the ability of *smy-8* and *smy-10* transgenes to ‘rescue’ the SL1 *trans*-splicing reporter assay phenotype associated with loss of the 11 endogenous *smy* genes (Figure 4). In animals carrying the wild-type *smy-8* transgene, we obtained reproducible reductions in intestinal GFP fluorescence consistent with restoration of SL1 *trans*-splicing. In contrast, the wild-type *smy-10* transgene was not able to affect intestinal fluorescence levels. These results further demonstrate that *smy* gene function contributes to SL1 *trans*-splicing activity.

Our data provide the first direct experimental support for the previously inferred function of the SmY RNAs in spliced leader *trans*-splicing. However, the molecular function of the SmY RNAs remained unclear. Given their association with SNA-3 and SUT-1, it is likely that they contribute to whatever role is played by these factors. We therefore further investigated the RIP-Seq data to determine the nature of the other transcripts associated with SNA-3 and SUT-1.

### SNA-3 and SUT-1 are associated with the 5′ ends of RNA polymerase II transcripts

**Figure 5.**
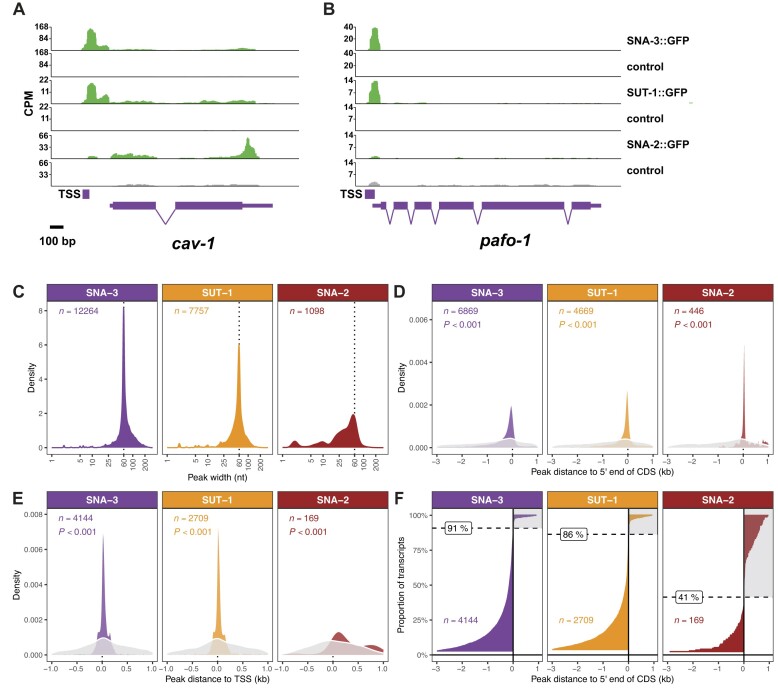
SNA-3 and SUT-1 are recruited to the 5′ ends of RNA polymerase II transcripts. (A, B) RIP-Seq read depth at the *cav-1* locus, a gene that produces SL1 *trans*-spliced transcripts (**A**), compared to *pafo-*1, which generates non-*trans*-spliced transcripts (**B**). ‘TSS’ indicates the location of transcription start sites. (**C**) SNA-3/SUT-1/SNA-2 RIP-Seq peak width distributions. (**D**) Distributions of distances between the SNA-3/SUT-1/SNA-2 RIP-Seq peaks and the 5′ end of the closest coding sequence (CDS). For clarity, only peaks located between −3000 (upstream) and 1000 nucleotides (downstream) of the CDS 5′ end are shown. (**E**) Distributions of distances between the SNA-3/SUT-1/SNA-2 RIP-Seq peaks and TSSs. Only peaks found between −1000 (upstream) and 1000 nucleotides (downstream) of TSS are shown for clarity. The grey density curves in panels D and E represent distances derived from equivalent numbers of peaks drawn at random across the genome (P value is from Kolmogorov-Smirnov test of equality of distributions). (**F**) Rank ordering of SNA-3, SUT-1 and SNA-2 RIP-Seq peaks to 5′ end of coding region distances, using only peaks from panel E. Peaks located >3000 nucleotides upstream are omitted for clarity. The dotted horizontal line represents the boundary for transcripts where the peak is upstream of the CDS (91%/86%/41%, respectively). The shaded grey areas of the plot marks transcripts with peaks that are downstream of the 5′ end of the CDS. This latter set of transcripts likely corresponds to those that are not spliced leader *trans*-spliced.

Manual inspection of the RIP-Seq read coverage at randomly chosen genes revealed for SNA-3::GFP and SUT-1::GFP, but not for SNA-2::GFP or GFP::SNA-1 (15), clusters of reads that are specifically associated with the 5′ ends of RNA polymerase II transcripts (see representative examples, Figure 5A, B). These read clusters tended to be around 60 nucleotides wide and, where the data was available, coincided with known transcription start sites (TSSs). For most genes, the RIP-Seq read peaks were upstream of the annotated 5′ end of the transcripts, which is consistent with the fact that the 5′ ends of most transcripts are removed through SL1 *trans*-splicing (Figure 5A). By the same reasoning, for genes that produce non-*trans*-spliced transcripts, such as *pafo-1* (Figure 5B), the peaks overlap the annotated 5′ end of the transcript.

The association of SNA-3 and SUT-1 with the 5′ ends of RNA polymerase II transcripts is consistent with our data showing that these proteins interact with the CBC-ARS2 complex (Figure 1B) (15), suggesting that these interactions may act to recruit SNA-3/SUT-1 to nascent RNA polymerase II transcripts (15). Strikingly, the presence of read clusters is independent of whether the gene is SL1 *trans*-spliced or not, raising the possibility that all RNA polymerase II transcripts are associated with SNA-3 and SUT-1 (Figure 5A, B).

To test this hypothesis genome-wide, we systematically identified genomic regions with enriched read coverage (read peaks) compared to control libraries and examined relative read peak distances to 5′ ends of transcripts and known TSSs. We identified 42,963 SNA-3 and 23,165 SUT-1 read peaks at FDR-corrected *P* ≤ 0.05. The analysis of the distribution of all read peak widths shows that the vast majority of peaks are about 60 nucleotides wide. In addition, a smaller number of reads form a minor approximately 25 nucleotides wide peak (Supplementary Figure S2A). However, many of these read peaks are likely to be biologically insignificant because of low read depth (as low as 10 reads) causing chance enrichment, and/or low enrichment score (as low as 1.01), despite statistically significance. Indeed, after removing RIP-Seq peaks with fewer than 20 reads or with enrichment scores of less than 5, the peak width distribution showed only a single about 60 nucleotides wide peak, suggesting that this filtered set of 12264 SNA-3 read peaks and 7757 SUT-1 read peaks is consistent with our initial visual inspections (Figure 5C). In contrast, we identified only 3978 statistically significant SNA-2 read peaks, of which only 1098 remained after filtering. These read peaks were also typically 60 nucleotides wide but had a much wider distribution skewed towards much narrower peaks (Figure 5C).

While the pattern of SNA-3 and SUT-1 read peaks is consistent with recruitment to the transcript 5′ end, possibly via the CBC-ARS2 complex, we wanted to investigate whether there was any evidence for *cis*-acting sequence motifs acting in the recruitment of these proteins. Sequence similarity clustering of the nucleotide sequences of all peaks that were 50–70 nucleotides wide indicated high sequence diversity (94.4% and 96.6% singleton clusters at 50% similarity for SNA-3 and SUT-1 respectively), which suggests that the recruitment is not dependent on a specific sequence binding motif. The same was observed for SNA-2, with 92.6% singleton clusters.

To globally determine the location of the SNA-3/SUT-1 RIP-Seq peaks, we examined the distances between a filtered set of read peaks (read depth ≥ 20 reads, enrichment score ≥ 5, peak width 30–90 nucleotides) and the 5′ end (the annotated AUG) of the coding sequence (CDS) of the nearest transcript. These distances were extremely variable (ranging from −97 057 nucleotides upstream to 47 123 nucleotides downstream of the CDS start), indicating that some read peaks are not located near 5′ ends of known transcripts. However, the most frequently observed distances placed the peaks upstream of CDS 5′ ends (−89 nucleotides for SNA-3 and -27 nucleotides for SUT-1) such that 70–72% of peaks (SNA-3: 6869 peaks; SUT-1: 4669 peaks) were no more than 3000 nucleotides upstream or 1000 nucleotides downstream of the CDS start of a transcript (Figure 5D). The distributions of peak distances were significantly different from those of the same numbers of random peaks drawn from across the genome (random peak distributions considered only the location of the peak summits, Kolmogorov–Smirnov tests, *D* = 0.25; *P* < 0.001 for SNA-3 and *D* = 0.31; *P* < 0.001 for SUT-1), indicating a true bias of read peaks being in close proximity to 5′ transcript ends. In contrast, we observed one order of magnitude fewer SNA-2 peaks that satisfied our filtering criteria (Figure 5D), and those peaks were typically located 15 nucleotides downstream of CDS 5′ ends rather than the upstream locations we observed for SNA-3 and SUT-1.

To explicitly test whether SNA-3 and SUT-1 peaks are associated with transcription start sites, we examined transcripts for which we had both TSS information and an overlapping or upstream RIP-Seq peak. We obtained 26,548 TSSs from the literature (Supplementary Table S5), of which 85% are correctly located upstream of the mRNA and CDS annotations of the associated transcript (15% overlap the mRNA or the CDS annotation), and 98% are no more than 5,000 nucleotides upstream of the CDS start. For 4,742 transcripts, we had both TSSs and SNA-3 RIP-Seq peaks, while we had TSSs and SUT-1 RIP-Seq peaks for 3,079 transcripts. The distances between RIP-Seq peak and TSSs were extremely variable (−41,979 to 10,793 nucleotides), indicating that some peak/TSS associations are biologically implausible (Supplementary Figure S2B). However, the most frequently observed distance between the RIP-Seq peaks and the TSSs was 23 nucleotides for SNA-3 and 0 nucleotides for SUT-1, and the absolute distance was no greater than 1000 nucleotides for 87% of the SNA-3 and 88% of the SUT-1 transcripts, indicating a large degree of coincidence of RIP-Seq peaks and TSSs (Figure 5E). As above, distances between TSSs and read peaks randomly drawn from across the genome were much more variable (Kolmogorov–Smirnov tests: *D* = 0.30; *P* < 0.001 for SNA-3 and *D* = 0.28; *P* < 0.001 for SUT-1), indicating a true tendency of RIP-Seq peaks and TSSs to coincide (Figure 5E), as predicted if SNA-3 and SUT-1 are recruited to the 5′ ends of RNA polymerase II transcripts. In contrast, we had only 226 transcripts with TSSs and SNA-2 peaks, of which 169 (75%) had RIP-Seq peaks at most 1000 nucleotides distant from the TSS. The distribution of these distances was skewed towards much larger downstream distances than those of SNA-3 and SUT-1, with a mode of 50 nucleotides (Figure 5E).

Our manual analysis of RIP-Seq coverage showed that for genes known to be non-*trans*-spliced, the SNA-3/SUT-1 RIP-Seq peaks overlapped the 5′ end of the CDS (Figure 5B), which is expected since the 5′ ends of these nascent transcripts correspond to the 5′ ends of the mature mRNAs on which the CDS annotations are based. In contrast, we found that for those genes that are *trans*-spliced, the RIP-Seq peaks were generally upstream and not overlapping the CDS, consistent with the fact that the 5′ ends of the nascent transcripts produced by such genes are removed during production of the mRNA. To confirm this pattern genome-wide, we examined the degree to which the RIP-Seq peaks that are near a TSS overlap with the first CDS of the transcript. Of the 4144 (SNA-3) and 2709 (SUT-1) peaks that were no more than 1000 nucleotides away from a TSS, 91% and 86% were upstream of the 5′ end of the CDS, whereas 9% and 14% were downstream of the 5′ end of the CDS (Figure 5F). These numbers are roughly consistent with the proportion of *trans*-spliced genes (85 – 87%) determined from genome-wide studies (8,46). Out of 3764 and 2340 genes where we predicted *trans*-splicing because the respective SNA-3 or SUT-1 peak was upstream of the 5′ end of the CDS (Figure 5F), 75.0% (SNA-3) and 63.0% (SUT-1) had a *bona fide trans*-splice acceptor site upstream of the 5′ end of the CDS. In contrast, among the 380 and 369 genes that we predicted not to be *trans*-spliced (Figure 5F), significantly fewer genes had a *bona fide trans*-splice acceptor site: 26.3% for SNA-3 (*X*^2^_1_ = 478.62; *P* < 0.001) and 28.2% for SUT-1 (*X*^2^_1_ = 190.84; *P* < 0.001). Although our predictions are bound to have misclassified some genes, these proportions indicate a clear tendency for *trans*-splicing status to be predictable by location of SNA-3 or SUT-1 RIP-seq peaks. In contrast, among the 169 transcripts with SNA-2 peaks only 69 (41%) would be predicted to be *trans*-spliced but there was no difference in the proportions of *bona fide trans*-spliced transcripts among the 69 predicted and the remaining 100 transcripts (70.0% versus 71.0%; *X*^2^_1_ = 0.04; *P* = 0.839). This indicates that SNA-2 peaks have no predictive power of *trans*-splicing status.

We verified that our analysis correctly associates peaks and TSSs with transcripts by manually examining 81 genes that are expected to be expressed in embryos (Supplementary Table S4). Among these genes, the distance between the TSS and the SNA-3 peak ranged from −208 bp to 265 bp with two outliers at 540 bp and 892 bp; these two cases were visually confirmed to be correct assuming the TSS annotation is correct. All genes had an upstream SNA-3 peak (distance range to 5′ end of CDS: −6,602 bp to −23 bp), suggesting that all transcripts are spliced leader *trans*-spliced (Supplementary Table S4). Seven outlier genes where the SNA-3 peak was more than 1 kbp upstream of the CDS were visually confirmed to be correct, suggesting that our algorithm is robust.

Overall, the read peak numbers, distribution patterns and peak profiles for the SNA-3 and SUT-1 RIP-Seq are distinct from those of SNA-2, further reinforcing that these two proteins form a pool distinct from the SL1 snRNP. Our data show that SNA-3 and SUT-1 are recruited to the 5′ ends of nascent RNA polymerase II transcripts, most likely through their affinities to the CBC-ARS2 complex and its associated factors. This places them at a key location, which, based on their affinities with components of the SL1 snRNP, makes them ideal candidates for factors involved in the recruitment of the SL1 snRNP to target pre-mRNAs.

### Depletion of SNA-3 impairs the interaction of SNA-2 with spliceosomal proteins

**Figure 6.**
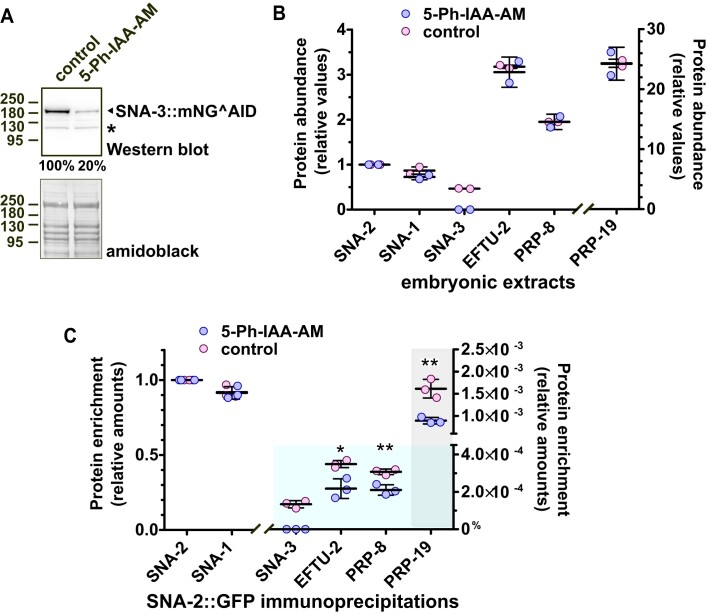
SNA-3 mediates the interaction between the SL1 snRNP and core spliceosome components. Embryos expressing auxin-inducible degron/mNeonGreen-tagged SNA-3 (SNA-3::mNG^AID) and GFP::SNA-2 were treated with the auxin analogue 5-Ph-IAA-AM or DMSO (control) before preparation of embryonic extracts. (**A**) Western blot of extracts prepared from 5-Ph-IAA-AM and control-treated embryos probed with anti-mNeonGreen antibody (upper panel) and then stained with amidoblack (lower panel). The relative abundances of SNA-3::mNG^AID are shown below the upper panel, standardised with respect to the amidoblack stain loading control. Asterisk indicates a non-specific signal. (**B**) Analysis of 5-Ph-IAA-AM and control-treated extracts by LC-MS/MS followed by label-free quantification. The graph shows the relative protein abundance of selected proteins determined using the total protein approach (30) and normalised with respect to SNA-2 protein, which was set to 1 (2 replicates each). (**C**) Analysis of immunoprecipitations performed on 5-Ph-IAA-AM and control-treated embryonic extracts using anti-GFP nanobodies coupled to agarose beads. Immunoprecipitations were performed in triplicate. Shown is the enrichment of selected proteins determined using the total protein approach (30) and normalized with respect to SNA-2 protein, which was set to 1. Enrichment of SNA-2 and SNA-1 is plotted on the left y-axis, and enrichment of SNA-3, EFTU-2, PRP-8 an PRP-19 is plotted on the right Y-axis (* *P*< 0.05; ** *P*< 0.01; *t*-test).

If SNA-3 has a role in recruitment of the SL1 snRNP to its target pre-mRNAs, we would expect that loss of SNA-3 would impact the formation of the SL1 snRNP-containing spliceosome. To investigate this, we tested whether the selective depletion of SNA-3 protein affects the interaction of SNA-2 with specific splicing factors. We tagged the endogenous *sna-3* gene with an auxin-inducible degron fused to mNeonGreen (*sna-3::mNG^AID*) via genome engineering. By treating embryos with the synthetic auxin 5-Ph-IAA-AM (26) prior to extract preparation, we were able to reduce embryonic SNA-3::mNG^AID protein levels 5-fold compared to control-treatment, as judged by Western blotting (Figure 6A). Analysis of protein content of the extracts by LC-MS/MS confirmed the reduction of SNA-3::mNG^AID in response to the treatment with 5Ph-IAA-AM: SNA-3 levels in extracts from 5-Ph-IAA-AM treated embryos were reduced below the level of detection. In contrast, SL1 snNP components SNA-1 and SNA-2, and splicing factors EFTU-2, PRP-8 and PRP19 were not affected (Figure 6B).

We then tested the effect of SNA-3 depletion on the interaction of the SL1 snRNP with the spliceosome by comparing proteins that coimmunoprecipitate with SNA-2::GFP from extracts from 5Ph-IAA-AM- and control-treated embryos (Figure 6C). While depletion of SNA-3 did not affect the co-immunoprecipitation of SNA-1 or of the Sm proteins (Supplementary Figure S3), the amount of SNA-3 was as expected reduced to below detectable levels. EFTU-2, PRP-8 and PRP-19 were the only splicing factors that were detected in all three immunoprecipitations performed with control-treated extracts. The amounts of these proteins were significantly reduced in immunoprecipitations from extracts where SNA-3 was depleted compared to extract from control-treated embryos. Importantly, none of these proteins were detected in control immunoprecipitations with beads only (Supplementary Table S6). This indicates that SNA-3, presumably as a component of the SmY snRNP, is required for the interaction of the SL1 snRNP with the spliceosome.

Note, SNRP-200 detected in the SNA-2::GFP immunoprecipitation in Figure 1 was also present at low levels in these immunoprecipitations. SNRP-200 protein abundance tended to be lower in immunoprecipitations from 5-Ph-IAA-AM treated extracts, but the difference was not statistically significant. EFTU-2 and PRP-19 are not shown in the SNA-2::GFP immunoprecipitation in Figure 1B. The co-immunoprecipitation of these proteins observed in Figure 6 was however not surprising. PRP-19 was earlier discovered in immunoprecipitations of GFP::SNA-1 (15). EFTU-2 was also detected in GFP::SNA-1 immunoprecipitations, but was not included in the final analysis as the label-free quantitation algorithm found one enrichment to be non-significant. In the SNA-2::GFP immunoprecipitations in Figure 1, EFTU-2 and PRP-19 were not included (Figure 1) as they were not detected in all immunoprecipitations. Together, this is compatible with subtle differences in low abundance proteins resulting in differences in enriched proteins between experiments. Importantly, PRP-8 was consistently and significantly enriched in all immunoprecipitations of SNA-1 and SNA-2, and the clear reduction of PRP-8 when SNA-3 was depleted is paralleled by the reduction of PRP-19 and EFTU-2 (Figure 6C).

PRP-8 and EFTU-2 are components of the U4/U6.U5 tri-snRNP and PRP-19 is a component of the NineTeen complex. During *cis*-splicing, they are components of the catalytic spliceosome, with PRP-8 at the core of the catalytic centre. Thus, these proteins serve as indicators for the interaction of the SL1 snRNP with the catalytic spliceosome.

In conclusion, the reduction of these proteins in SNA-2 immunoprecipitations when SNA-3 is depleted, indicates that SNA-3 protein is involved in the interaction between the SL1 snRNP and the spliceosome, consistent with a role for the SmY snRNP in recruiting the SL1 snRNP to its target pre-mRNAs.

## Discussion

Spliced leader *trans*-splicing has been studied for more than three decades. From the earliest studies, a key challenge was understanding the mechanism by which the spliced leader RNA and its target pre-mRNA are brought together to enable the assembly of the spliceosome. We directly address this challenge here, as well as providing insight into previous seminal discoveries in nematode spliced leader *trans*-splicing: the role of SUT-1 protein and the SmY RNA family (6,13).

SmY RNAs have been implicated in spliced leader *trans*-splicing since their discovery but direct evidence supporting this inference has been lacking. Our work fills this knowledge gap, directly showing the involvement of SmY RNAs in SL1 *trans*-splicing, using our reporter gene and qRT-PCR assays that monitor this process. Consistent with a role in spliced leader *trans*-splicing, we show that SNA-3, a highly conserved, essential protein involved in this process (15), co-immunoprecipitates SmY RNAs, as does its binding partner SUT-1, a protein similarly implicated in SL1 *trans*-splicing (13–15). Consistent with being important components of spliced leader *trans*-splicing, animals lacking SmY RNAs arrest as early larvae.

Since they are novel in terms of both structure and sequence composition, the mechanistic function of the SmY RNAs is unclear. Previous workers have suggested that they function in Sm protein recycling after completion of a spliced leader *trans*-splicing reaction (13). Their model involved base-pairing between specific SmY RNAs and SL1 and SL2 RNAs, with the SmY RNAs predicted to base pair with SL2 RNAs, diversifying with their targets. This model is inconsistent with our data. Firstly, we show that loss of ten of the twelve *smy* genes is tolerated, at least under laboratory culture. Moreover, a single SmY RNA, SmY-8, can functionally substitute for the other eleven SmY RNA, while the proposed SL1-specific SmY RNA, SmY-10, cannot. Furthermore, SmY-10 appears to be functionally distinct from the other SmY RNAs, since it is not able to interact with either SNA-3 or SUT-1 as assayed by RIP-Seq analysis, despite being able to interact with Sm proteins. Its function is also dispensable, at least in terms of viability.

The function of the SmY RNAs is likely to be associated with their protein partners, SNA-3 and SUT-1. However, the role of these two proteins has hitherto been similarly obscure. The RIP-Seq data presented here suggested a possible function for SNA-3 and SUT-1 associated with the 5′ ends of RNA polymerase II transcripts. These RNA interactions are consistent with other interactions we observed for the two proteins, both in this current study and in previous work (15), showing that they associate with components of the CBC-ARS2 complex, which co-transcriptionally binds to the cap of nascent RNA polymerase II transcripts. The localization of these factors at the 5′ ends of pre-mRNAs places them at a location that would be suitable for the recruitment of the SL1 snRNP, a role that is consistent with their known interactions, and one that we have previously proposed (15). Whether the SmY RNAs are similarly recruited to the 5′ ends of nascent transcripts remains to be determined, but their interactions with SNA-3 and SUT-1 suggest they have an important role either in the recruitment of SNA-3/SUT-1 to pre-mRNAs or in promoting their functions at this location.

**Figure 7.**
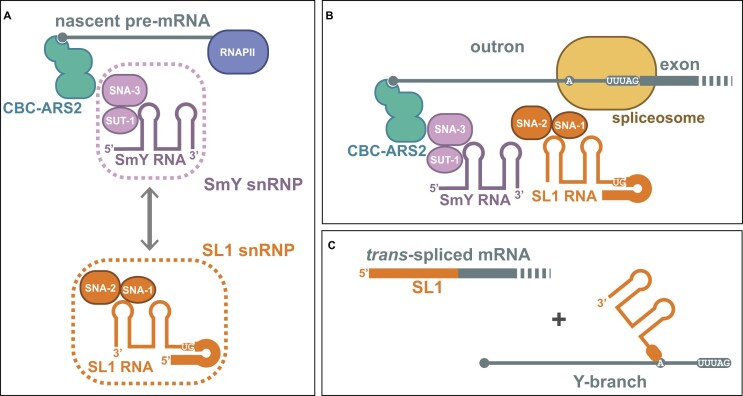
Model for assembly of the nematode *trans*-spliceosome. (**A**) SNA-3 and SUT-1 proteins, likely as components of the SmY snRNP, interact with the 5′ ends of nascent pre-mRNAs, marked by the CBC-ARS2 complex, and form transient associations with the SL1 snRNP (shown are components SL1 RNA and SNA-1 and SNA-2 proteins) (**B**) This interaction is stabilised by interactions with spliceosome components assembled at the branchpoint and 3′ splice site of the outron, and allows the formation of the *trans*-spliceosome, which catalyses the formation of mature *trans*-spliced mRNA and a Y-branch by-product that is enzymatically degraded (**C**).

It is striking that SNA-3/SUT-1 are associated with pre-mRNAs regardless of whether they are SL1 *trans*-spliced. This suggests either that they have additional functions independent of *trans*-splicing and/or they are not the sole determinants involved in the assembly of the *trans*-spliceosome around the nascent transcripts. We cannot rule out the former possibility, but the two explanations are also not mutually exclusive. Taking our cues from the mechanistic basis of U1 snRNP function in *cis*-spliceosomal assembly (47), we propose that SNA-3 and SUT-1 are recruited to the 5′ ends of all pre-mRNAs, and are thus able to recruit the SL1 snRNP through formation of short-lived and transient complexes (Figure 7). Our model proposes that additional interactions are required to form more stable complexes. Recruitment of SL1 snRNP to nascent transcripts would not be productive unless the growing transcript contained a *bona fide* branchpoint and 3′ splice site. These would recruit spliceosome components, starting with SF1 and U2AF2, and initiate the formation of the spliceosome. Interaction of the SL1 snRNP with spliceosome components, as for example observed between the *Ascaris* homologue of SNA-2 and SF1 (Denker 2002), would thus stabilize the SNA-3-mediated interaction between SL1 snRNP and its target pre-mRNA. This model predicts that loss of SNA-3 should interfere with the interaction of the SL1 snRNP with the spliceosome, and we have shown that this is the case - depletion of SNA-3 impairs the interactions between SNA-2 and components of the catalytic spliceosome (Figure 6).

We have known for some time that spliced leader *trans*-splicing is mechanistically similar to *cis*-splicing (5,6,48), but that mechanisms must have evolved to facilitate the assembly of the spliced leader and its target pre-mRNA into the spliceosome. Our work has significantly increased our understanding of how this might be achieved for nematode SL1 *trans*-splicing. However, the precise mechanistic details are still unknown, specifically, we do not understand how the individual components achieve what they do. Except for SNA-2, which possesses RRM domains, we do not know much about the functional elements of the protein components involved beyond what amino acid sequence conservation tells us. The important next steps in understanding this widespread eukaryotic RNA processing event will be defining its precise biochemistry. We are now well placed to begin this analysis.

## Supplementary Material

gkae321_Supplemental_Files

## Data Availability

The mass spectrometry proteomics data have been deposited to the ProteomeXchange Consortium via the PRIDE partner repository with the dataset identifier PXD040352 (see also Supplementary Table S2). RIP-Seq data have been deposited in the ArrayExpress database at EMBL-EBI (www.ebi.ac.uk/arrayexpress) under accession number E-MTAB-13193 (see also Supplementary Table S3).

## Notes

Present address: Rotimi Fasimoye, University of Dundee School of Life Sciences, Medical Research Council Protein Phosphorylation Unit, Sir James Black Building, Dundee DD1 5EH, UK.
