# Supplementary material for: A nematode-specific ribonucleoprotein complex mediates interactions between the major nematode spliced leader snRNP and its target pre-mRNAs
Source: Nucleic Acids Res. 2024 Apr 27;52(12):7245–60. doi: 10.1093/nar/gkae321 (PMC11229312; doi:10.1093/nar/gkae321)
Supplement: gkae321_Supplemental_Files [file gkae321_supplemental_files.zip › Eijlers et al Supplementary_Methods_Data_Legends.docx]

**Eiljers et al Supplementary Material**

**Supplementary Methods**

**Preparation of *C. elegans* embryo extracts**Synchronised populations were grown in liquid culture in S-complete medium supplemented with OP50. Approximately 1.5 x 10^6^ embryos were transferred into a 2-litre flask containing 500 ml S-complete medium (50 mM potassium phosphate buffer (pH 6), 100 mM NaCl, 10 mM potassium citrate (pH 6), 3 mM CaCl_2_, 3 mM MgSO_4_, 13 µM cholesterol and 1x standard trace metal solution) and grown overnight at 20°C in a shaking incubator at 200 rpm to L1 stage. After hatching of the embryos (normally after 12 – 24 hours), 14 – 16 g frozen OP50 bacteria were added, and the incubation (200 rpm, 20°C) was continued for 80 – 90 h until synchronous gravid adult worms emerged. Adults were recovered by centrifugation at 400x g for 2 min, washed using MilliQ water, floated on 1 M sucrose and then washed again using MilliQ water to remove debris.

Animals were then lysed by alkaline hypochlorite treatment and embryos recovered by floating on sucrose. Normally, embryos were washed three times with MilliQ water, except for embryos that were subjected to 5-Ph-IAA-AM or DMSO control-treatment. These embryos were washed three times with M9 buffer before dilution with 6.7 volumes of M9 buffer and treated for 1h at room temperature with 50 µM 5-Ph-IAA-AM using a stock solution of 50 mM 5-Ph-IAA-AM (TOCRIS) dissolved in DMSO or control-treated with 0.1 % DMSO. Then all embryos were washed twice in 10mM Tris-HCl (pH 8.0), 1.5 mM MgCl_2_, 10 mM KCl, 50 mM sucrose, 0.05 % NP-40, and once in homogenisation buffer (10mM Tris-HCl (pH 8.0), 1.5 mM MgCl_2_, 10 mM KCl, 1 mM DTT, 50 mM sucrose, 0.05 % NP-40, 1X cOmplete EDTA-free protease inhibitor (Roche 1873560) and 1mM PMSF) and finally resuspended in 3 volumes homogenisation buffer. Embryos were lysed using a Dura-Grind Dounce Tissue Grinder (Wheaton) and extracts were then cleared by 10 min centrifugation at 10,000 × g at 4°C followed by two times 5 min centrifugation at 10,000 × g at 4°C. The final supernatants were dialysed against 20 mM Tris-HCl (pH 8.0), 50 mM KCl, 1 mM DTT, 10% glycerol, 0.5 mM EDTA, 1 mM PMSF at 4°C, and then flash-frozen in liquid nitrogen for storage at -80°C in an ultra-low temperature freezer.

**Immunoprecipitation of proteins**
Anti-GFP nanobody and control agarose (Chromotek GmbH)were washed three times in wash buffer (10 mM Tris-HCl (pH8), 60 mM KCl, 4 mM MgCl_2_, 5% glycerol, 0.25 mM EDTA, 3.5 mM DTT, 0.5 mM PMSF). Extracts were supplemented with ¼ volume of 175 mM KCl, 20 mM MgCl_2._ Where indicated, extracts were treated with 5 units/ml RNase A and 200 units/ml RNase T1 (Ambion RNAse Cocktail (Invitrogen)) for 2 h at 4°C. Normally, for each immunoprecipitation, 5 µl of settled agarose beads were mixed with 250 µl of extract and incubated on a rotator at 4°C. After 1 h, the beads were collected by centrifugation at 2000x g for 2 min at 4°C and washed twice by resuspension in 500 µl of 20 mM Tris-HCl (pH8.0), 100 mM KCl, 0.1 mM EDTA, 0.1% NP-40, 1 mM DTT followed by centrifugation at 1000x g for 1 min at 4°C. Then they were transferred into fresh 1.5 ml tubes and washed two more times with the same buffer. After removal of the final wash, the wet beads were flash frozen in liquid nitrogen and stored at -80°C.

**Proteomic analysis by LC-MS/MS**Proteins bound to beads were resuspended in 100 µl 50 mM ammonium bicarbonate, reduced in 4 mM dithiothreitol for 25 min at 60°C, S-alkylated in 8 mM iodoacetamide for 30 min at 25⁰C in the dark, and then digested with 0.2 µg porcine trypsin (Promega sequencing grade) overnight at 37°C. The reaction was stopped by freezing at -70⁰C and drying by vacuum centrifugation. Peptides were dissolved in 40 µl 0.1% trifluoroacetic acid, desalted using µ-C18 ZipTips (Merck Millipore) and eluted in 70% acetonitrile, 0.1% trifluoroacetic acid. After drying by vacuum centrifugation, the peptides were dissolved in 10 µl 0.1% trifluoroacetic acid then analysed by LC-MS/MS using a Q Exactive Plus/Ultimate 3000 RSLCnano system (Thermo Scientific). Following pre-concentration on a trapping column (C18 PepMap 100), the peptides were reverse-flushed to the nano column (PepMap RSLC C18) and separated using a gradient of acetonitrile in water containing 0.1% formic acid. A “Top 10” Full MS/Data-dependent MS2 method was used to acquire mass data during 60 min (1) . MS1 scans (375-1750 m/z) had a resolution of 70,000, automatic gain control of 3e6, and Maximum IT of 50 ms. Ions with charge states of +2 to +5 were selected for higher-energy collisional dissociation (NCE of 28%). MS2 scans (variable first mass) had a resolution of 17,500, AGC of 5e4, and Maximum IT of 100 ms. Dynamic exclusion was set at 40 s, and peptide ions were preferred.

Protein composition of embryonic extracts was determined using 10 µg extract made up with 50 mM ammonium bicarbonate to 100 µl. Reduction with 4 mM dithiothreitol and S-alkylation with 8 mM iodoacetamide and treatment with trypsin were done as described above. Dried samples were dissolved in 10 µl 0.1% trifluoroacetic acid and analysed by LC-MS/MS as described above.

**Differential protein abundance analysis**Raw data files were processed using Maxquant version 1.6.5.0 (2) using the *C. elegans* reference proteome UP000001940 downloaded on 24/08/2021. Carbamidomethylation of cysteine was used as fixed modification; oxidation of methionine and acetylation of the protein N terminus were set as variable modifications. Instrument selection was Orbitrap and Trypsin/P cleavage specificity was used. The minimal peptide length was 7 amino acids and a maximum of two missed cleavages were allowed. FTMS MS/MS match tolerance was set to 10 ppm. False discovery rate was 1% for peptide and protein identifications. Each protein group was required to contain at least two unique or razor peptides, with each peptide used only once for protein identification (Razor protein FDR). For label-free quantification (LFQ) (3), the LFQ minimum ratio count was set to two and both unique and razor peptides were used for quantification. The match from and to option was used as Match type. Data were analysed using Perseus software (version 1.6.5.0) as described (4, 5). Proteins missing valid LFQ values in any of the immunoprecipitations with anti-GFP nanobody coupled agarose beads were excluded. The LFQ intensities were log2-transformed and missing values were replaced with low abundance values based on simulated normal distribution using Perseus standard settings. Then, the differences between proteins amounts in immunoprecipitations with anti-GFP nanobody beads and protein amounts in the control precipitations with agarose beads were examined using a two-sample t-test with a permutation-based FDR. Data were visualised using volcano plots with a conservative q-value *<*0.05 and an s0 value of 1.8 to highlight clearly enriched proteins. Volcano plots were drawn using GraphPad Prism version 5 for Windows, GraphPad Software, San Diego, California US ([www.graphpad.com](http://www.graphpad.com)). Heatmap was produced using Excel (Microsoft).

**RNA immunoprecipitation sequencing (RIP-Seq)**Immunoprecipitations were performed in quadruplicate using either anti-GFP nanobody coupled agarose beads or control agarose beads as described previously for protein immunoprecipitations (5), except that 500 µl extracts supplemented with ¼ volume of 175 mM KCl, 20 mM MgCl_2_ and 10 µl settled agarose beads were used. Immunoprecipitations and wash steps were done as described, and washed beads were treated with 200 µl 0.2 mg/ml Proteinase K in 20 mM Tris-HCl (pH7.5), 1% SDS, and 1 mM EDTA at 65°C for 15 min.

The RNA was recovered by two subsequent extractions with acidic phenol:chloroform:isoamylalcohol (125:24:1, pH 4.5). The final aqueous phase was supplemented with 10 µg linear acrylamide , 1/10 volume 3M sodium acetate (pH 5.2) and 2.5 volumes of ethanol and the RNA collected by centrifugation, , washed with 70% ethanol and air dried.

Library preparation, sequencing, and analysis were done at the Centre for Genome Enabled Biology and Medicine (CGEBM; University of Aberdeen). Libraries were prepared using the Diagenode D-Plex Small RNA-seq Kit for Illumina, following the manufacturer’s instructions, and sequenced on an Illumina NextSeq500 high-output v2.5 flow cell, producing 76-bp single end reads. Illumina adapters, as well as technical sequences detailed in the D-Plex Small RNA manual (polyA/T tails; template-switching motif; CATS adapters), were removed from the data using CUTADAPT 2.3 (7). Poor-quality bases with a phred score below 20 were trimmed and reads shorter than 15 bp after trimming were discarded. The quality-filtered reads were aligned to the *C. elegans* genome (ENSEMBL WBcel235 assembly) using HISAT 2.1.0 (8) and quantified against gene annotations using FEATURECOUNTS 1.6.2 (9). Multi-mapping reads were assigned to all mapping locations and each location received a fractional count. Gene-based read counts were analysed in R using the DESeq2 V1.26 package (10). Differential gene expression between each IP experiment and its matched control samples was identified with negative binomial models, shrinking fold changes of low-count genes with the *apeglm* method (11) and correcting P-values for multiple testing using the false-discovery rate (FDR) method (12). Genes of interest (snoRNAs, snRNAs and ncRNAs) were identified using the WormMine tool. Volcano plots were drawn using GraphPad Prism version 5 for Windows, GraphPad Software, San Diego, California US ([www.graphpad.com](http://www.graphpad.com)).

1. Bateman,N.W., Goulding,S.P., Shulman,N.J., Gadok,A.K., Szumlinski,K.K., MacCoss,M.J. and Wu,C.C. (2014) Maximizing peptide identification events in proteomic workflows using data-dependent acquisition (DDA). *Mol. Cell. Proteomics*, **13**, 329–338.

2. Cox,J. and Mann,M. (2008) MaxQuant enables high peptide identification rates, individualized ppb-range mass accuracies and proteome-wide protein quantification. *Nat. Biotechnol.*, **26**, 1367.

3. Cox,J., Hein,M.Y., Luber,C.A., Paron,I., Nagaraj,N. and Mann,M. (2014) Accurate proteome-wide label-free quantification by delayed normalization and maximal peptide ratio extraction, termed MaxLFQ. *Mol. Cell. Proteomics*, **13**, 2513–2526.

4. Tyanova,S., Temu,T., Sinitcyn,P., Carlson,A., Hein,M.Y., Geiger,T., Mann,M. and Cox,J. (2016) The Perseus computational platform for comprehensive analysis of (prote)omics data. *Nat. Methods*, **13**, 731–740.

5. Fasimoye,R.Y., Spencer,R.E.B., Soto-Martin,E., Eijlers,P., Elmassoudi,H., Brivio,S., Mangana,C., Sabele,V., Rechtorikova,R., Wenzel,M., *et al.* (2022) A novel, essential trans-splicing protein connects the nematode SL1 snRNP to the CBC-ARS2 complex. *Nucleic Acids Res.*, **50**, 7591–7607.

6. Wiśniewski,J.R., Hein,M.Y., Cox,J. and Mann,M. (2014) A “proteomic ruler” for protein copy number and concentration estimation without spike-in standards. *Mol. Cell. Proteomics*, **13**, 3497–3506.

7. Martin,M. (2011) Cutadapt removes adapter sequences from high-throughput sequencing reads. *EMBnet. journal*, **17**, 10.

8. Kim,D., Paggi,J.M., Park,C., Bennett,C. and Salzberg,S.L. (2019) Graph-based genome alignment and genotyping with HISAT2 and HISAT-genotype. *Nat. Biotechnol.*, **37**, 907–915.

9. Liao,Y., Smyth,G.K. and Shi,W. (2014) featureCounts: an efficient general purpose program for assigning sequence reads to genomic features. *Bioinformatics*, **30**, 923–930.

10. Love,M.I., Huber,W. and Anders,S. (2014) Moderated estimation of fold change and dispersion for RNA-seq data with DESeq2. *Genome Biol.*, **15**, 550.

11. Zhu,A., Ibrahim,J.G. and Love,M.I. (2019) Heavy-tailed prior distributions for sequence count data: removing the noise and preserving large differences. *Bioinformatics*, **35**, 2084–2092.

12. Benjamini,Y. and Hochberg,Y. (1995) Controlling the false discovery rate: A practical and powerful approach to multiple testing. *J. R. Stat. Soc. Series B Stat. Methodol.*, **57**, 289–300.

**Supplementary Figures**

**
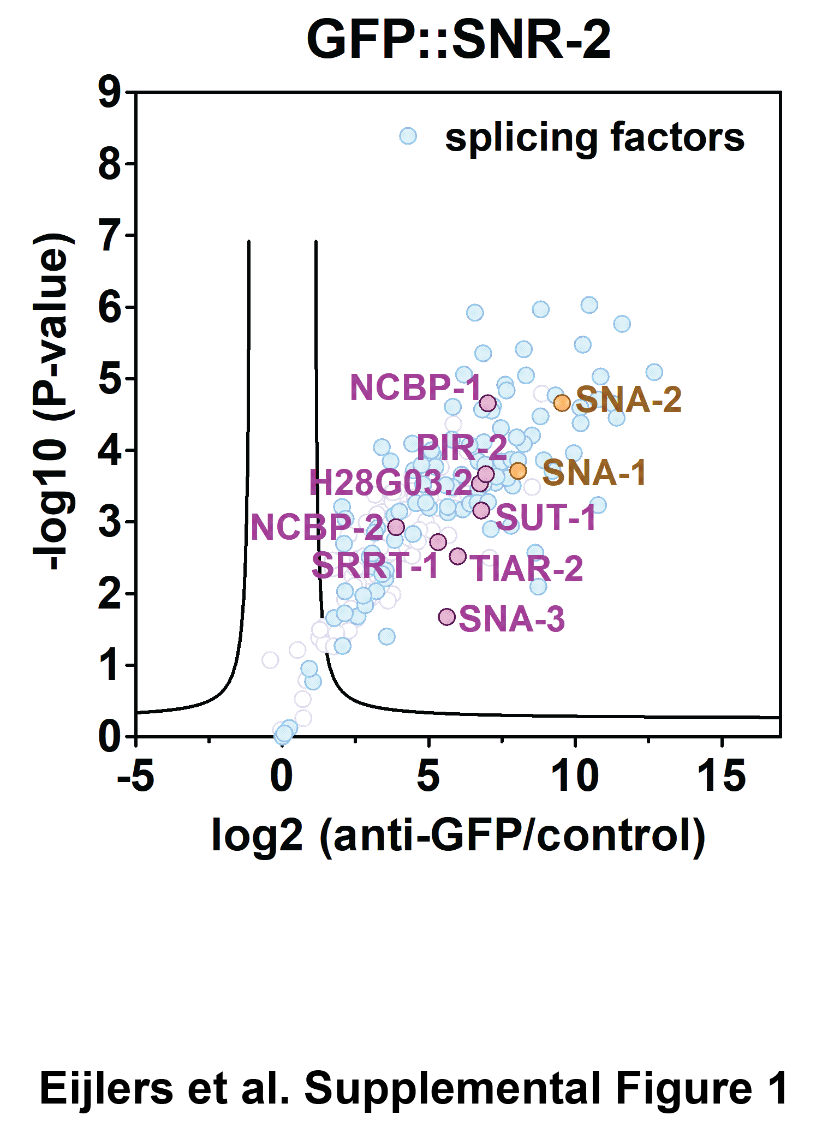
**

**Supplementary Figure 1. SNR-2/SmB protein interactions.** Proteins enriched by co-immunoprecipitation with GFP::SNR-2. Components of the spliceosome are indicated in blue, while proteins enriched in SNA-3 immunoprecipitations are indicated in magenta. SNA-1 and SNA-2, specific components of the SL1 snRNP, are in gold. All other proteins are unshaded.

**
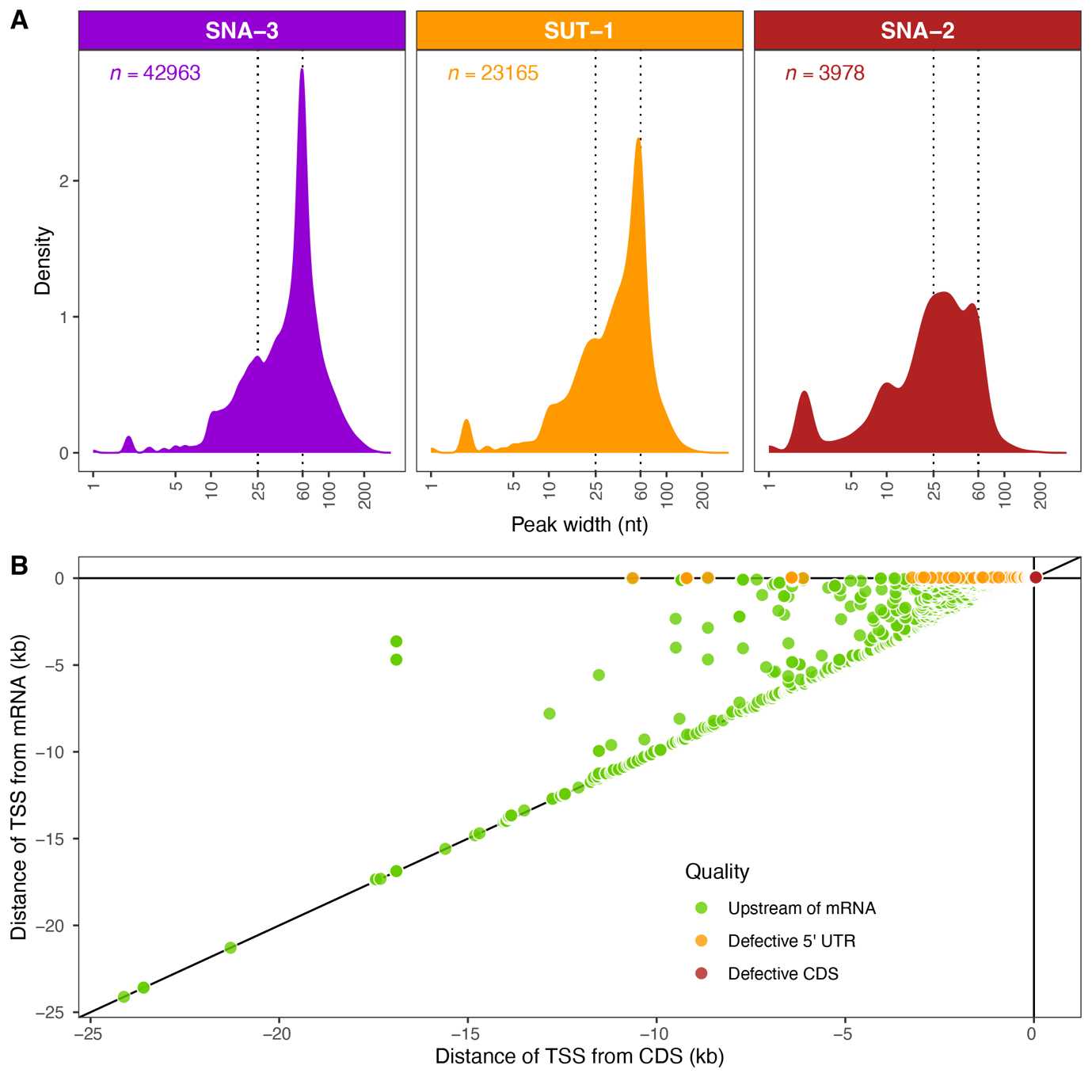
**

**Supplementary Figure 2: Summary of features of SNA-3 and SUT-1 RIP-Seq peaks, transcription start sites (TSSs) and 5’ transcript ends. (A**) Distributions of peak widths (logarithmic scale) of all identified peaks. (**B**) Relationship between TSS distances to 5’ end of CDS and mRNA annotations of transcripts. Good quality TSSs are expected to be upstream of both 5’ end of each CDS and mRNA annotations; overlap into the mRNA or CDS annotations indicates defective 5’ UTR or CDS annotations.

**
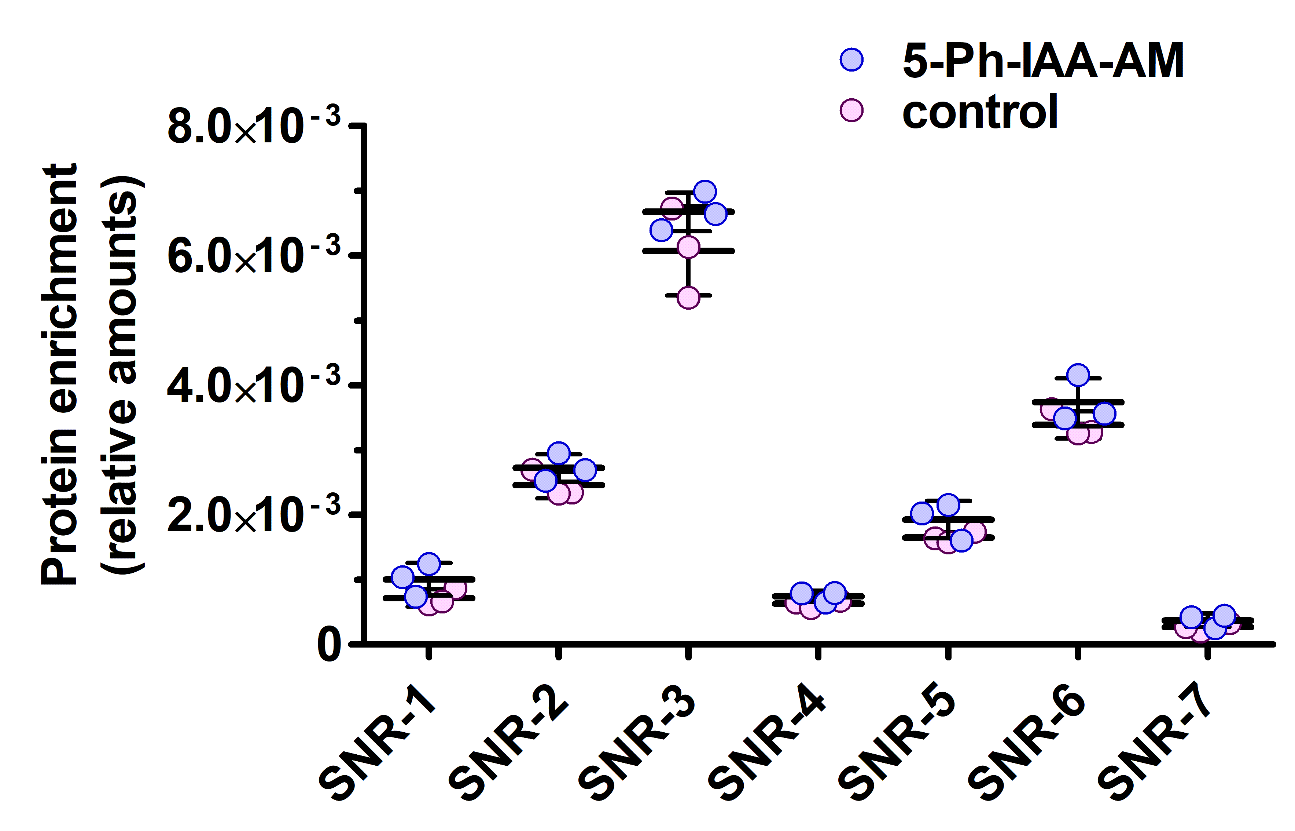
Supplementary Figure 3: Depletion of SNA-3 does not affect the co-immunoprecipitation of Sm proteins with SNA-2.** Analysis of immunoprecipitations performed on extracts prepared from PE1220 (SNA-3::AID::mNG expressing) embryos treated with/without 5-Ph-IAA-AM using anti-GFP nanobodies coupled to agarose beads as described in Figure 6 legend. Shown are the levels of Sm proteins (SNR-1 to SNR-7) recovered.

**Supplementary Table Captions**

**Supplementary Table 1: Oligonucleotide sequences used to generate plasmid constructs as outlined in Materials and Methods.** These include oligonucleotides used to (i) generate guide RNA expression plasmids to make *smy* gene deletions, (ii) detect wild type and mutant *smy* alleles, and the size of their amplicons, (iii) generate *smy* gene rescuing plasmids, and (iv) generate guide RNA expression plasmids and homology repair templates used to make the *sna-2* GFP knock-in allele.

**Supplementary Table 2: Identification and quantitation of proteins interacting with GFP-tagged proteins.** Perseus data used to create Figure 1 and Supplementary Figure 1. Worksheets detail the updated gene/protein annotations based on recent gene naming assignments, and the results for each GFP-tagged protein, including the *C. elegans* strain used in each case.

**Supplementary Table 3: RIP-Seq identification of RNAs associated with GFP-tagged proteins.** DESeq2 statistics on which Figure 2 and Supplementary Figure 1 are based. Transcripts are listed by WormBase gene identifier (WB), gene name (Gene) and, where appropriate, non-coding RNA type (Name and Group, respectively). Worksheets give the results for each GFP-tagged protein and are also listed by the respective strain used.

**Supplementary Table 4: Verification of SNA-3 RIP-Seq peak detection algorithm using a set of well-characterised embryonic genes with defined transcription start sites (TSSs).** Detailed explanatory legend is presented within the Excel file.

**Supplementary Table 5: Genomic coordinates of transcription start sites (TSSs) and SNA-3 and SUT-1 RIP-Seq read peak clusters.** Detailed explanatory legend is presented within the Excel file.

**Supplementary Table 6: Effect of SNA-3::AID::mNG depletion on proteins interacting with SNA-2.** Detailed explanatory legend is presented within the Excel file.
